# From Consultation to Collaboration: A Patient-Centered Approach to Shingles Pain and Postherpetic Neuralgia Management

**DOI:** 10.3390/jpm15050191

**Published:** 2025-05-08

**Authors:** Yin-Tse Wu, Hsuan-Chih Lao, Sheng-Chin Kao, Ying-Chun Lin, Ying-Wei Yang, Ying-Hsin Li, Yi-Jun Chen

**Affiliations:** 1 Department of Medicine, MacKay Medical University, New Taipei City 252005, Taiwan; yinzewu0908@gmail.com (Y.-T.W.); sckao1974@gmail.com (S.-C.K.); elegant.beaver@gmail.com (Y.-C.L.); doc5577k1972@yahoo.com.tw (Y.-W.Y.); 2 Department of Anesthesiology, MacKay Memorial Hospital, Taipei 104217, Taiwanshina.b889@mmh.org.tw (Y.-J.C.); 3 Institute of Brain Science, College of Medicine, National Yang Ming Chiao Tung University, Taipei 112304, Taiwan

**Keywords:** shingles pain, postherpetic neuralgia, shared decision-making, intervention therapy, pain control

## Abstract

**Background/Objectives**: Herpes zoster (shingles), caused by reactivation of the varicella zoster virus, often leads to acute pain that may progress to postherpetic neuralgia (PHN). Current evidence is insufficient to determine the optimal interventional treatment for these conditions. This study aimed to evaluate the effectiveness of shared decision-making (SDM) forms developed by MacKay Memorial Hospital (MMH) in reducing patient anxiety and improving personalized care. **Method**: Between 1 August 2022 and 30 August 2024, we retrospectively reviewed SDM records of patients with shingles pain and PHN who were referred to the pain clinic for interventional treatment due to unresolved pain. The SDM forms were developed, reviewed, and authorized by the MMH Committee of Medical Quality and Safety. We analyzed the chosen interventions, anxiety levels, pain intensity, and patient preferences regarding treatment selection. **Results**: A total of 51 individuals (36 with shingles pain, 15 with PHN) were included in this cohort study. Most patients with acute or chronic zoster pain opted for subcutaneous steroid injections. Anxiety scores significantly decreased following SDM intervention, from 5.0 (IQR: 3.5–5.0) to 3.0 (IQR: 2.0–3.0) in shingles patients and from 5.0 (IQR: 4.0–5.0) to 2.0 (IQR: 2.0–3.0) in PHN patients. Pain intensity, measured using the numerical rating scale (NRS), also improved markedly after interventional pain management, with scores reducing from 8.0 (IQR: 6.0–9.0) to 3.0 (IQR: 1.0–6.5) in shingles patients and from 5.0 (IQR: 4.0–8.0) to 2.0 (IQR: 1.0–3.0) in PHN patients. Shingles patients expressed greater concern about the risks of interventional therapy complications, whereas PHN patients prioritized cost, complication rates, treatment frequency, and continuity of care. Additionally, SDM forms received high scores for promoting patient participation and knowledge, indicating that they improved their understanding of their condition and treatment options. **Conclusions**: SDM significantly improved patient comprehension, reduced anxiety, facilitated informed treatment decisions, and strengthened doctor–patient communication for those with shingles pain and PHN.

## 1. Introduction

Herpes zoster [1], also known as shingles, is a viral infection caused by the reactivation of the varicella zoster virus (VZV). In Taiwan, the estimated lifetime risk of herpes zoster is 32.2%, with an incidence of 4.97 cases per 1000 person-years. Women have a higher incidence rate than men (5.20 versus 4.72 cases per 1000 person-years) [2]. The incidence increased significantly with age, with approximately two-thirds of cases occurring in adults over 40 years old [3]. Similar trends are observed in Western countries [1,3,4]. After recovering from chickenpox, the virus remains dormant in the body, typically in nerve tissues, and can reactivate years or even decades later, causing shingles. This condition is characterized by a painful rash, usually localized to one side of the body or face, often appearing as a stripe of blisters. Herpes zoster can lead to chronic, potentially lifelong complications, particularly in older adults, with postherpetic neuralgia being the most common.

Postherpetic neuralgia (PHN) [1,5] is defined as pain that persists for more than three months from the initial onset of the skin rash. Its incidence rate occurs in approximately 20% of patients with herpes zoster [5]. The most commonly affected areas by postherpetic neuralgia include the thoracic spine, cervical spine, and trigeminal nerve [6]. The pain can be burning, sharp, or stabbing and may be intermittent or constant, significantly impacting daily activities and quality of life. Therefore, managing and relieving the pain is the primary goal for patients.

Currently, individualized pain management for both acute and chronic pain from herpes zoster can be divided into four main categories [5,7]: oral medication (e.g., gabapentinoids, tricyclic antidepressants), lidocaine patches, injections (e.g., peripheral nerve blocks, subcutaneous steroid or botox injections), and neuromodulation (e.g., pulsed radiofrequency, spinal cord stimulation). However, the current evidence is insufficient for determining the best single interventional treatment [7]. Therefore, it becomes even more important to discuss all the available treatment options with the patient.

Shared decision-making (SDM) is a collaborative healthcare process where providers and patients make informed treatment decisions together. Through this process, the patient’s values, preferences, and circumstances are considered alongside the medical knowledge and expertise of the healthcare provider. The goal is to ensure that patients are well-informed, actively involved, and empowered to make decisions about their care [8,9].

Currently, to our knowledge, there have been no relevant studies discussing whether SDM is helpful in treatment choices and outcomes for patients with shingles pain and PHN. Therefore, this study aimed to examine the therapeutic and communicated benefits of the shared decision-making forms developed by Mackay Memorial Hospital, a tertiary care teaching hospital, to engage patients and establish personalized medical care.

## 2. Methods

### 2.1. Development of the Shared Decision-Making (SDM) Forms

To develop the most suitable health education forms, we reviewed past literature [7] and conducted in-hospital surveys to better understand what patients want to know about the disease and the latest treatment options. All descriptions and evidence-based information have been reviewed and authorized by the Committee of Medical Quality and Safety. The content of SDM forms is divided into three main sections (detailed information is provided in Supplemental Materials S1 and S2):

1. Introduction to acute neuralgia from herpes zoster and postherpetic neuralgia–providing essential information about the conditions.
2. Introduction and comparison of relevant interventional treatments—covering options such as local steroid injection, pulsed radiofrequency, paravertebral block, and peripheral nerve stimulation.
3. Patient considerations—addressing factors such as cost, potential complications, and the possibility of hospitalization.

### 2.2. Enrollment

All patients in this study were recruited from Mackay Memorial Hospital in Taiwan, and all SDM forms completed from 1 August 2022 to 30 August 2024 were included in this study. Clinical intervention options and treatment effectiveness were reviewed through medical records. Patients diagnosed with shingles pain or postherpetic neuralgia who were referred to the pain clinic were included in the study, whereas those who did not complete the SDM form were excluded. Patients were categorized into two groups based on the duration of their pain: those with pain lasting less than 90 days were classified under the shingles pain group, while those with pain persisting more than 3 months were classified under the PHN group.

**Figure 1:**
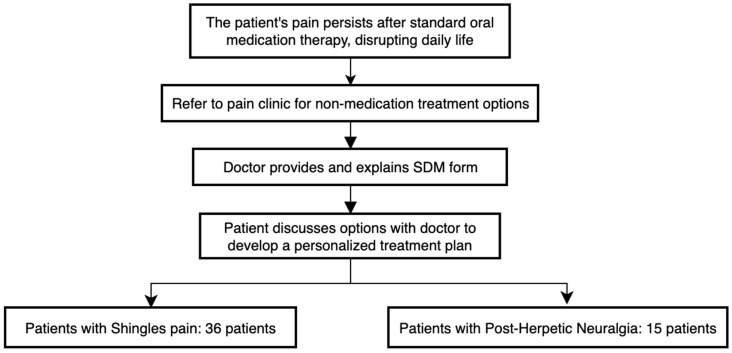
Flow chart of shared decision-making.

In our medical referral routine (Figure 1), any patient with shingles pain or postherpetic neuralgia whose pain symptoms do not significantly decrease after standard oral medication therapies and continue to cause substantial disruption to daily life will be referred to the pain clinic to explore treatment options beyond medication. At the pain clinic, after completing a medial review and physical examination, patients will be provided with a shared decision-making (SDM) form titled “My postherpetic neuralgia remains quite painful despite medication. What interventional treatment options do I have?” or “The blisters from my shingles have healed a long time ago, yet I’m still in pain. What interventional treatments can I consider?” Following this, a nurse will conduct a one-on-one professional counseling session lasting approximately 30 min, to assist the patient in completing the questionnaire, during which the available interventional treatment options and their respective advantages and disadvantages will be outlined. The patient will then discuss these options with the physician to develop a personalized treatment plan.

### 2.3. Consistency Among Staff

The staff in charge of the SDM illustration comprised two nurses and three pain physicians from the outpatient pain clinic. With guidance and supervision from the three physicians of the pain division, a consensus conference was conducted to confirm the implementation procedure and methods to practice the instructions for using auxiliary tools and to achieve consistent expression among all staff.

### 2.4. Outcome Measures

The primary outcome of this study is to evaluate whether the patient’s anxiety level can be reduced after SDM. The secondary aims include assessing the distribution of patient considerations, the initial numerical rating scale (NRS) of pain, which refers to the assessment conducted before SDM, and the helpfulness scores for participation and understanding. Additionally, the treatment options chosen by the patients and the change of postinterventional NRS, which refers to the final recorded assessment after the last treatment, will be reviewed.

### 2.5. Assessment

The patient’s level of anxiety and helpfulness scores are assessed based on a 5-point scoring method, with “very much” scored as 5 points, “relatively much” as 4 points, “some” as 3 points, “a little” as 2 points, and “totally not” as 1 point.

The patient’s considerations are assessed based on a 6-point scoring method, with “very much” scored as 5 points, and “totally not” as 0 points. Considering the different disease progression between PHN and shingles pain, patients with PHN tend to be more about the number of repetitive treatments. Therefore, adjustments were made to the SDM regarding patients’ consideration. Among patients in the shingles pain group, their identified considerations were categorized into five areas: waiting time for treatment, cost, complication risk, number and frequency of treatments, and the potential need for hospitalization. In contrast, patients in the PHN group identified considerations in six areas: waiting time for treatment, cost, complication rate, number and frequency of treatments, continuity of treatment, and the possibility of hospitalization.

### 2.6. Statistical Analysis

The results were presented as numbers and percentages for categorical data and as median with quartile deviation for continuous data. Continuous variables were analyzed using t-tests or the Wilcoxon two-sample test, as appropriate. For the comparison of changes in anxiety and pain intensity (NRS), paired *t*-tests were used to assess intragroup differences between baseline and post-intervention, including post-SDM illustration or after pain management, provided that normality assumptions were met. If normality assumptions were not met, the Wilcoxon rank-sum test was applied for paired data analysis. Statistical analyses were performed using STATA 15 software (StataCorp. 2017. Stata Statistical Software: Release 15. College Station, TX, USA: StataCorp LLC), and a *p*-value of less than 0.05 was considered statistically significant.

All figures were created using the Prism software (version 8.9.1), and a flow chart was created using draw.io online software (version 26.0.10).

## 3. Results

### 3.1. Demographic Data of the Patients

A total of 51 patients were enrolled in the study, including 36 (70.6%) in the shingles pain group and 15 (29.4%) in the postherpetic neuralgia (PHN) group (Table 1). The median age was 71.5 years for shingles patients and 75.0 years for PHN patients. Female patients accounted for 52.8% of the shingles pain group, whereas male patients comprised 53.3% of the PHN group. All participants were of Asian ethnicity.

The thoracic region was the most commonly affected dermatome in both groups (56.4% in the shingles pain and 64.7% in PHN), followed by cervical involvement in the shingles pain group (28.2%) and sacral involvement in the PHN group (17.6%). In terms of comorbidities, hyperlipidemia (19.4% vs. 26.7%), type 2 diabetes mellitus (13.9% vs. 20.0%), and hypertension (25.0% vs. 13.3%) were frequently observed in the shingles pain and PHN groups, respectively.

### 3.2. Patients with Shingles Pain

**Figure 2:**
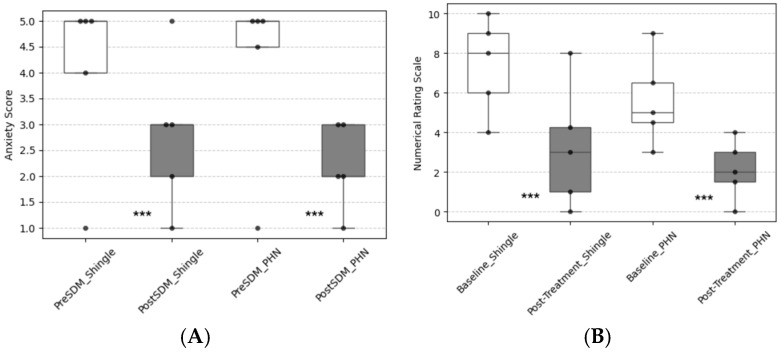
(**A**) Difference in patient’s anxiety level; (**B**) difference in patient’s NRS score (**A**). The anxiety levels of patients with shingles pain and PHN significantly decreased after SDM, with reductions of 3.0 and 2.0, respectively. *** The difference in anxiety score shows a significant decrease (*p*-value < 0.05) by paired *t*-tests. (**B**) The NRS scores of patients with shingles pain and PHN significantly decreased after SDM, with both groups showing a reduction of 3.0. *** The difference in NRS shows a significant decrease (*p*-value < 0.05) by the Wilcoxon rank-sum test.

A total of 36 patients with shingles pain completed the shared decision-making (SDM) form during the study period (Table 1). This cohort included 17 males and 19 females, with a median age of 71.5 years. Prior to the SDM process, patients exhibited an anxiety level of 5.0 (IQR: 3.5–5.0), which decreased to 2.0 (IQR: 2.0–3.0) following SDM (Figure 2A), representing a significant reduction of 3.0 points (*p*-value < 0.05). The treatment process considerations identified by these patients were categorized into five areas: waiting time for treatment, cost, complication risk, number and frequency of treatments, and the potential need for hospitalization. Notably, the greatest concern was the complication risk, with a score of 5.0 (IQR: 5.0–5.0) (Table 2). The helpfulness scores for participation and understanding were both 5.0 (IQR: 4.0–5.0) (Table 2).

All patients with shingles pain received at least one treatment modality, which included oral medications, peripheral nerve blocks, or pulsed radiofrequency (PRF), with ten patients receiving more than one intervention therapy. Most patients (47.2%) opted for conservative pain management, such as oral analgesics or lidocaine patches, while 30.6% of patients chose subcutaneous steroid injection as their first treatment modality following SDM completion. For those who chose interventional pain management, different interventions might be administered, such as starting with a subcutaneous steroid injection, followed by paravertebral steroid injection if needed. Only one patient chose PRF neuromodulation for the shingles pain immediately after SDM, while two other patients received PRF following steroid and local anesthetics injection at the affected dorsal root ganglion. The baseline NRS score was 8.0 (IQR: 6.0–8.0), greatly decreasing to an average post-interventional score of 3.0 (IQR: 1.0–6.5; *p*-value < 0.05) (Figure 2B).

### 3.3. Patients with Postherpetic Neuralgia

Fifteen patients, including 8 males and 7 females, with postherpetic neuralgia and a median age of 75.0 years, were enrolled (Table 1). The duration of PHN varied among patients, ranging from 6 months to 6 years. The initial anxiety score averaged 5.0 (IQR: 4.0–5.0) before the implementation of SDM and improved to 2.0 (IQR: 2.0–3.0) afterward (Figure 2A), reflecting a reduction of 2.0 points (*p*-value < 0.05). We identified six primary concerns regarding the treatment process for these patients: waiting time for treatment, cost, complication rate, number and frequency of treatments, continuity of treatment, and the possibility of hospitalization. Among these, the most critical concerns were cost, complication rate, number and frequency of treatments, and continuity of treatment, with a median score of 5.0 (IQR: 4.5–5.0) (Table 2). Their helpfulness scores for participation and understanding were both 5.0 (IQR: 4.5–5.0) and 5.0 (IQR: 4.0–5.0), respectively (Table 2).

Compared to the patients with shingles pain, 73.3% of PHN patients preferred conservative pain management after SDM completion. Upon completion of the treatment course, nerve blocks (40.0%) and PRF neuromodulation (33.3%) were more commonly used in the PHN group. In contrast, only 13.0% received subcutaneous steroid injections, which were more common in the shingles group. During treatment, the baseline NRS score for these patients was 5.0 (IQR: 4.0–8.0), which significantly decreased to 2.0 (IQR: 1.0–3.0) post-intervention (*p*-value < 0.001), representing a reduction in pain intensity of 3.0 points after treatment (Figure 2B).

## 4. Discussion

In our study, both the NRS score and anxiety score after SDM significantly decreased (*p*-value < 0.05). Patients with shingles pain were more concerned about the complication risk of intervention therapy, while those with PHN prioritized complication rates, treatment frequency, and continuity of treatment. High SDM helpfulness scores for participation and knowledge indicate that the SDM form is beneficial for improving patients’ understanding of disease and involvement in treatment decisions.

The main risk factor for both herpes zoster and PHN is age [10,11,12]. This may be related to the decline in cell-mediated immunity specific to the varicella zoster virus as aging. Additionally, in Asian countries, the incidence of herpes zoster and PHN is higher in females than in males [3,11,13], though the mechanisms are not yet clear. Our median age was 73.0 years, with 51% of patients being female, aligning with existing demographic studies.

Shared decision-making (SDM) combines evidence-based medicine with collaborative communication, integrating physician expertise with patient preferences to select appropriate therapies [8,9]. Our SDM forms gather patient consideration, enabling healthcare providers to understand what matters most to each individual. SDM has been shown to improve patients’ anxiety levels and pain control. Chiu et al. [14] found that anxiety decreased from 3.5 points to 2.9 points (*p*-value < 0.001) in patients with atrial fibrillation using SDM, echoing similar results reported by Cheng et al. [15]. For pain management, Rajshri Bolson et al. [16] found that the SDM-tool group achieved equivalent or better pain control while requiring fewer opioid prescriptions compared to the control group. Our study also observed a reduction in anxiety levels, with decreases of 3.0 and 2.0 points (*p*-value < 0.01) in patients with shingles pain and PHN, respectively, reinforcing the benefits of SDM in helping patients gain clarity about their situation. Additionally, therapeutic effectiveness improved, as reflected by reductions in NRS scores of 3.0 and 3.0 points (*p*-value < 0.05) in patients with shingles pain and PHN, respectively. These improvements followed SDM implementation in patients who experienced limited relief from previous medications and were referred by other specialists.

Current treatments [5] for PHN typically begin with the application of 5% lidocaine patches. If these are ineffective, gabapentinoids, amitriptyline, or even weak opioids may be added. Should oral medicines fail to alleviate pain, interventional treatments are then considered. Taking into account factors such as invasiveness, cost, and safety, first-line intervention treatments for PHN include the subcutaneous injection of botulinum toxin A or triamcinolone, while the second-line options consist of paravertebral block and pulsed radiofrequency [7]. If severe pain persists despite these interventions, spinal cord stimulation could be considered [7]. However, current evidence remains insufficient to determine the optimal interventional single treatment for PHN [7]. At this juncture, SDM plays a critical role in guiding treatment decisions.

While SDM promotes patient involvement and autonomy in medical decision-making, cultural and educational factors in many Asian countries often result in more passive patient roles [17]. In Western countries, patients tend to express their preferences but still generally expect physicians to make the final decision [18]. Therefore, future efforts should focus on strategies to enhance patient engagement in decision-making beyond relying solely on physicians’ directives.

The long-term psychological impact of SDM, particularly regarding anxiety reduction and overall well-being, warrants further investigation. While our study did not collect long-term follow-up data, previous study supports SDM’s role in improving mental health and treatment adherence [19,20]. For example, Van Roosmalen et al. [21] explored the effectiveness of SDM for BRCA1/2 mutation carriers considering screening versus prophylactic surgery. Their SDM group reported fewer intrusive cancer-related thoughts and improved general health nine months later, while the control group showed no such improvements, highlighting SDM’s potential to positively influence long-term psychological outcomes.

Furthermore, demographic factors such as gender and socioeconomic status may influence SDM’s effectiveness. Brown et al. [22] found that SDM significantly reduced chronic pain in females compared to males. Other studies indicate that patients with higher education levels are more engaged in decision-making [23], and females aged 60–69 perceived greater clinician engagement in SDM than male patients of the same age [24]. These findings highlight the importance of considering patient characteristics when designing and implementing SDM strategies.

Our study has several limitations. First, some variables, such as anxiety levels and NRS scores, relied on patient self-reports. These measurements may have been influenced by patients’ expectations or their desire to demonstrate improvement after receiving personalized care. This reliance on subjective measures may limit objectivity and may not fully reflect actual changes in anxiety and pain. Second, patients participating in the SDM process may have been inherently more proactive or engaged with their care. This potential selection bias could have influenced anxiety reduction and perceived treatment outcomes, thereby limiting the generalizability of our findings. Third, two experienced nurses were responsible for explaining the SDM content. While both received thorough training and aimed to maintain consistency in their explanations, variations in healthcare providers’ communication styles were still evident in practice. This may have impacted patients’ understanding of the SDM content. Lastly, the relatively small sample size collected over the two-year period was primarily due to time constraints in clinical practice. With more than 20 patients scheduled during a half-day pain clinic, it was challenging to complete all steps of the SDM tools, particularly the assessment of anxiety. The limited sample size not only reduces the generalizability of our findings to the broader population but also increases the risk of type II errors, potentially obscuring subtle yet meaningful effects of SDM. Furthermore, the small number of participants in each group made it difficult to conduct meaningful subgroup analyses, limiting our ability to explore whether factors such as gender or the choice of interventional treatment might influence the effectiveness of SDM. Future studies should include a larger sample size to minimize bias and enhance the reliability of findings.

## 5. Conclusions

Our SDM approach demonstrated significant improvements in anxiety levels among patients with shingles pain and PHN while also facilitating more personalized treatment discussions. By encouraging patient engagement, SDM increases understanding of the pros and cons of various intervention therapies, supporting more informed and value-based decisions. On the physician’s side, it allows doctors to understand what matters most to the patient and provides them with ways to address those concerns, all of which contribute to improved doctor-patient communication. However, the long-term psychological impact of SDM, along with the factors that may influence its effectiveness, requires further evaluation in future studies with larger, randomized cohorts.

## Figures and Tables

**Table 1:** Demographic data of the patients.

Characteristic	SHINGLES PAIN	PHN
	(N = 36)	(N = 15)
**Age, median (QD)**	71.5 (59.5–75.0)	75.0 (70.5–81.0)
**Sex, n (%)**	36	15
Male	17 (47.2)	8 (53.3)
Female	19 (52.8)	7 (46.7)
**Race** **, n (%)**		
Asian	36(100)	15 (100)
**Duration of pain, median (QD)**	55.5 (35.0–76.0, days)	22.0 (14.0–40.0, months)
**The distribution of lesions, n (%)**		
C spine	11 (28.2)	1 (5.8)
T spine	22 (56.4)	11 (64.7)
L spine	3 (7.6)	2 (11.7)
S spine	3 (7.6)	3 (17.6)
**Comorbidities, n (%)**		
Type 2 diabetes mellitus	5 (13.9)	3 (20.0)
Hypertension	9 (25.0)	2 (13.3)
Chronic kidney disease	2 (5.6)	2 (13.3)
Hyperlipidemia	7 (19.4)	4 (26.7)
Cardiovascular disease	3 (8.3)	2 (13.3)
Cancer	7 (19.4)	2 (13.3)

Values are numbers (percentages) unless stated otherwise. Percentages might not total to 100 due to rounding. QD means quartile deviation, which is the difference between the 25th percentile (Q1) and the 75th percentile (Q3).

**Table 2:** Outcomes after SDM Implementation.

Group	Shingles Pain	PHN
	(N = 36)	(N = 15)
**Treatment received after SDM completion number (%)**		
Oral medications/local lidocaine patch	17 (47.2)	11 (73.3)
Subcutaneous steroid injection	11 (30.6)	2 (13.3)
Nerve block	6 (16.7)	2 (13.3)
Pulsed radiofrequency	1 (2.8)	0 (0.0)
**Treatment received after SDM number (%)**		
Oral medications/local lidocaine patch ^¶^	36 (100.0)	15 (100.0)
Subcutaneous steroid injection	13 (36.1)	2 (13.0)
Nerve block	12 (33.3)	6 (40.0)
Pulsed radiofrequency	3 (8.3)	5 (33.3)
Receiving more than one intervention therapy	10 (27.8)	3 (20.0)
**Primary outcome, median(QD)**		
Anxiety level (before)	5.0 (3.5–5.0)	5.0 (4.0–5.0)
Anxiety level (after)	3.0 (2.0–3.0)	2.0 (2.0–3.0)
**Secondary outcomes, median(QD)**		
NRS (before)	8.0 (6.0–9.0)	5.0 (4.0–8.0)
NRS (after)	3.0 (1.0–6.5)	2.0 (1.0–3.0)
Patient considerations		
Waiting time	5.0 (3.0–5.0)	5.0 (3.0–5.0)
Cost	5.0 (3.0–5.0)	5.0 (4.5–5.0)
Complication rate	5.0 (5.0–5.0)	5.0 (4.5–5.0)
Number and frequency of treatments	2.0 (5.0–5.0)	5.0 (4.5–5.0)
Hospitalization	3.0 (5.0–5.0)	5.0 (4.5–5.0)
Continuity of treatment	None	5.0 (4.5–5.0)
The helpfulness scores for participation	5.0 (4.0–5.0)	5.0 (4.5–5.0)
The helpfulness scores for knowing	5.0 (4.0–5.0)	5.0 (4.0–5.0)

Anxiety score and helpfulness SDM scores range from 1 to 5. Patient consideration range from 0 to 5. Numerical rating scale (NRS) ranges from 0 to 10. Percentages might not total to 100 due to rounding. QD means quartile deviation, which is the difference between the 25th percentile (Q1) and the 75th percentile (Q3). Anxiety level (before) and NRS (before) refers to the assessment conducted before SDM. Anxiety level (after) and NRS (after) refers to the final recorded assessment after the last treatment. ^¶^, oral medication was provided as a standard baseline treatment during the patient’s subsequent outpatient visit, regardless of whether they received interventional therapy.

## Data Availability

The data presented in this study are available on request from the corresponding author.

## Supplementary Materials

The following supporting information can be downloaded at: https://www.mdpi.com/article/10.3390/jpm15050191/s1, File S1: My postherpetic neuralgia remains quite painful despite medication. What interventional treatment options do I have? File S2: The blisters from my shingles have healed a long time ago, yet I’m still in pain. What interventional treatments can I consider?

## Abbreviations

The following abbreviations are used in this manuscript:

PHN	postherpetic neuralgia
SDM	shared decision making
NRS	numerical rating scale
