# Supplementary material for: From Consultation to Collaboration: A Patient-Centered Approach to Shingles Pain and Postherpetic Neuralgia Management
Source: J Pers Med. 2025 May 8;15(5):191. doi: 10.3390/jpm15050191 (PMC12112963; doi:10.3390/jpm15050191)
Supplement: Supplementary file 1 [file jpm-15-00191-s001.zip › jpm-3551056-Supplementary Material S2_English.pdf]

Supplementary Material S2

Introduction

This decision aid was designed to assist patients with postherpetic neuralgia (PHN) in understanding their treatment options, clarifying their values and preferences, and engaging in shared decision-making with their healthcare providers. The questionnaire includes four sections covering treatment-related concerns, decision-making support, evaluation of healthcare provider efforts, and demographic information.

Section 1. Treatment-Related Concerns

Please indicate how important each of the following factors is to you when considering interventional treatment options:

Item	Factor	Considerations<br>"Very much" scored as 5 points ←→"Totally not" as 0 point.
1-1	Waiting time	
1-2	Cost	
1-3	Risk of complications	
1-4	Number and frequency of treatments	
1-5	Continuity of treatment	
1-6	Need for hospitalization	

Section 2. Decision-Making Process Assistance

Please indicate whether the following statements apply to your experience using this decision aid:

Item	Statement
2-1	Helped you recognize that you need to make a decision
2-2	Helped you feel prepared to make a better decision
2-3	Helped you think about the advantages and disadvantages of each option
2-4	Helped you consider which advantages and disadvantages matter most to you

2-5	Helped you understand that this decision depends on what matters most to you
2-6	Helped you organize your thoughts regarding this decision
2-7	Helped you reflect on the extent to which you want to be involved in this decision
2-8	Helped you identify questions you would like to ask your healthcare provider
2-9	Helped you prepare to express to your healthcare provider what matters most to you
2-10	Helped you prepare for follow-up discussions with your healthcare provider
2-11	Please rate your level of anxiety regarding your current medical problem before using this decision aid (scale: 0–10)
2-12	Please rate your level of anxiety regarding your current medical problem after using this decision aid (scale: 0–10)
2-13	Were there any descriptions, images, formats, or questions in this decision aid that were difficult for you to understand or answer? (Yes/No)
2-14	Are there any important questions related to your decision-making that were not addressed in this decision aid? (Yes/No, please specify)

Section 3. Evaluation of Healthcare Provider’s Efforts

Please rate the healthcare provider’s effort on the following aspects:

Item	Question
3-1	How much effort did the healthcare provider make to help you understand your health problem? (Scale: 0–10)
3-2	How much effort did the healthcare provider make to listen to what matters most to you regarding your health problem? (Scale: 0–10)
3-3	How much effort did the healthcare provider make to include what matters most to you when deciding the next steps? (Scale: 0–10)

Section 4. Demographic Information

Item	Information
4-1	Identity

4-2	Age
4-3	Decision-maker (Patient, Family, Both)
4-4	Time of completion
